# Giant right atrial myxoma presented with symptoms of Budd–Chiari syndrome: a case report

**DOI:** 10.1097/MS9.0000000000001116

**Published:** 2023-07-31

**Authors:** Echreiva Med Sidi El Moctar, Chighaly El Hadj Sidi, Mohammed Abdulrazzak, Manar Ahmed Kamal, Mohamed Feissal Mohamed Ahmed, Khaled Boye

**Affiliations:** a Faculty of Medicine, Ain Shams University, Cairo; b Center National de Cardiology, Nouakchott, Mauritania; c Faculty of Medicine, University of Aleppo, Aleppo, Syrian Arab Republic; d Faculty of Medicine, Benha University, Benha, Egypt

**Keywords:** Budd–Chiari syndrome, case report, myxoma, right atria

## Abstract

**Introduction and importance:**

Right atrial myxoma is rarely associated with Budd–Chiari syndrome (BCS). In this paper, we present a case of a young patient with a giant right atrial myxoma complicated by the development of BCS.

**Case presentation:**

A 26-year-old female presented to the emergency room with persistent abdominal pain, ascites, lower limb edema, and an acute episode of dyspnea. Abdominal computed tomography revealed a lesion in the right cardiac cavity that resembled an intracardiac thrombus. Transthoracic echocardiography showed a large heterogeneous mass in the right atrium protruding into the right ventricle and a large thrombus interfering with inferior vena cava flow. The patient underwent cardiac surgery to remove the atrial mass, and histopathologic examinations confirmed the diagnosis of atrial myxoma.

**Clinical discussion:**

The right atrial myxoma is relatively rare, especially the giant ones. Rarely have intracardiac tumors such as giant right atrial myxoma been identified as a risk factor for the onset of BCS.

**Conclusion:**

In the differential diagnosis of BCS, right atrial tumors, including myxomas, should be considered, especially when other explanations are absent.

## Introduction

Highlights

- Right atrial myxoma is rarely associated with Budd–Chiari syndrome.
- The blockage of hepatic venous outflow and obstruction of the inferior vena cava are defining characteristics of the rare condition known as Budd–Chiari syndrome.
- A 26-year-old female presented to the emergency room. The patient was suffering from dyspnea with a history of persistent abdominal pain. There was no family history nor a medical history of a similar condition.
- The patient underwent emergency surgery, carried out under cardiopulmonary bypass with cardioplegia, while venous drainage was made by direct cannulation of the superior vena cava and the right common femoral vein.

The blockage of hepatic venous outflow and obstruction of the inferior vena cava (IVC) are defining characteristics of the rare condition known as Budd–Chiari syndrome (BCS). So, hepatic vein thrombosis or mechanical outflow restriction are the two main causes of BCS^1^. The risk factors for hepatic vein thrombosis include prothrombotic coagulation disorders and chronic myeloproliferative diseases^2^. The most frequent causes of mechanical outflow blockage in BCS are cancer and infections, which are frequently accompanied by obstruction of the hepatic section of the IVC^3^.

The most frequent primary cardiac tumors are myxomas. According to estimates, 20% of myxomas come from the right atrium, 5% come from both the atria and the ventricle, and more than 75% start in the left atrium, either at the mitral annulus or the fossa ovalis boundary of the interatrial septum^4,5^. Conversely, right atrial myxoma is relatively rare, especially giant ones^6^.

Rarely have intracardiac tumors such as giant right atrial myxoma been identified as a risk factor for the onset of BCS; nevertheless, surgical excision of these tumors is typically a curative procedure^7,8^. This paper shows a case of a young patient who developed BCS, due to complications from a giant right atrial myxoma, and highlights the challenges encountered in managing this unique case as per the Surgical CAse REport (SCARE) 2020 Guidelines^9^.

## Case presentation

A 26-year-old female presented to the emergency room on the 17th of May 2022. The patient was suffering from dyspnea with a history of persistent abdominal pain. There was no family history nor a medical history of a similar condition.

According to radiological investigations, echography confirmed ascites and hepatomegaly, and abdominal computed tomography (CT) revealed a huge mass in the right cavity of the heart as well as thrombus formation in the IVC. The patient was referred to a cardiology center for suspected intracardiac thrombosis.

Upon examination, oxygen saturation was 99%, respiratory rate 30 breaths per minute, pulse rate 100 beats per minute, blood pressure 112/82 mmHg, and temperature 37.5°C. Abdominal examination revealed marked abdominal distension accompanied by the palpable tender liver with lower limb edema, whereas cardiac examination was nonspecific with normal heart sounds and jugular veins.

Electrocardiogram exposed normal sinus rhythm with no abnormalities, while laboratory tests showed the following: white blood cell count of 4820/ml, hemoglobin of 7.6 mg/dl, platelets of 339 000/ml, fasting blood glucose of 72 mg/dl, urea of 0.31 g/l, creatinine 6 mg/l, CRP 35 mg/l, prothrombin time (PT) 56 s, international normalized ratio (INR) 6, and partial thromboplastin time (PTT) 100 s.

**Figure 1:**
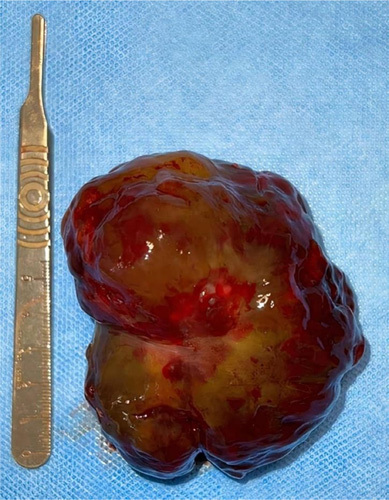
Heterogenous gelatinous mass.

**Figure 2:**
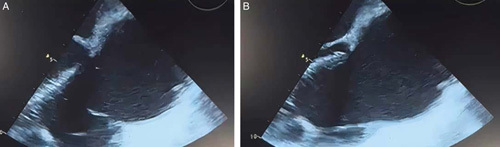
Huge myxoma occupying the right atria.

**Figure 3:**
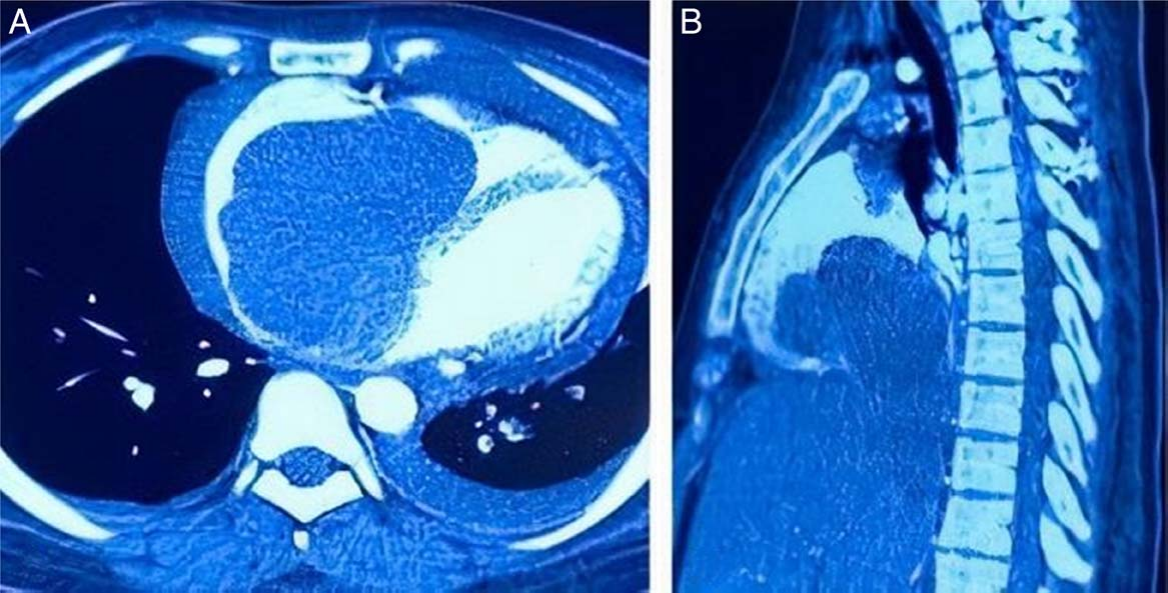
(A) Computed tomography scan shows a large mass in the right cavity of the heart. (B) The liver is congested and enlarged.

Transthoracic echography showed a bulky mass of heterogeneous appearance at the level of the right atrium and 2/3 of the right ventricle, as well as a thrombus that took the path of the IVC until it joined the right atrium, thoracic–abdominopelvic CT angiography, was performed to describe the lesion and search for an etiologic focus showed; a large mass in the right atrium measuring 93×66 mm extending toward the IVC, pulmonary embolism in the arterial lobar branches of the lower lobe of the left lung, in addition to pleuro-pericardial effusion with moderate ascites, and heterogeneous congested liver with marked dilation of the hepatic veins. There was no evidence of renal tumor or pelvic mass on CT (Figs. 1–3).

The patient underwent emergency surgery, carried out under cardiopulmonary bypass with cardioplegia, while venous drainage was made by direct cannulation of the superior vena cava, and the right common femoral vein. A large myxoma was removed, and its implantation base resected, followed by IVC embolectomy with a Fogarty probe. Furthermore, the tricuspid valve was tested before occlusion of the right atrium, and transesophageal echography revealed moderate regurgitation. Unfortunately, the patient died on the second postoperative day due to massive acute right ventricular failure.

## Discussion

The incidence of primary cardiac neoplasms is 1.38 per 100 000 people annually, most of which are myxomas^10^. Approximately 75% of myxomas are located in the left atrium, whereas right atrial myxomas account for around 25%^11^. Right atrial myxomas are rare, especially giant ones^6^; in a meta-analysis study involving more than 1000 individuals, only 13% were found to be right atrial myxomas. Therefore, they are usually not diagnosed until a serious disease such as pulmonary embolism or BCS develops^12^. In this case report, we discuss the case of a young patient with a giant right atrial myxoma complicated by the development of BCS.

Our patient presented with typical symptoms of BCS as abdominal pain, ascites, and lower limb edema, while Nina *et al*.’s^13^ study shows that myxomas are asymptomatic or manifest with constitutive symptoms such as fever, arthralgia, anemia, and weight loss.

Our case had a right atrial myxoma associated with thrombus formation in the supra-hepatic part of the IVC resulting in the development of BCS. In agreement with our findings, Anagnostopoulos *et al*.’s^7^ study describes a case of BCS and portal vein thrombosis caused by a right atrial myxoma. In contrast, a 49-year-old male patient reported by Kynta *et al*.^14^ presented with myxoma originating from the Eustachian valve without thromboembolic events. Unlike our patient, right atrial myxoma is present immediately at the junction of the IVC and the right atrium, associated with thrombus formation in IVC and pulmonary embolism as in Karimi *et al*.’s study^15^.

In our case, the patient had a benign myxoma and passed away after surgery because of developing acute heart failure, while some patients presented with a malignant tumor and died soon after being diagnosed^16^. In our case, systemic embolization was the main cause of postoperative mortality, while the previous literature showed that atrial arrhythmias and heart block were potential problems that could happen in the early or late postoperative period^17^.

To explain the unexpected death of our case, the development of tricuspid regurgitation could be one of the expected mechanisms. Firstly, the giant myxoma was pendulous in the right ventricle and prevented the tricuspid valve from closing, and this mass may form an obstruction for a long period. After the giant myxoma removing, the small ventricle was in front of a large volume of blood to accommodate (preload), causing it to expand acutely, and the tricuspid leak also increased preload, leading to a right ventricular failure^18^. The second mechanism could be associated with giant myxoma with pulmonary arterial hypertension (PAH)^19^, but this mechanism seems to be less involved because postoperative echocardiography did not objectify a lethal PAH (systolic pulmonary arterial pressure of 50 mmHg). On the other hand, a significant increase in the tricuspid leak, especially since the myxoma was extirpated without crumbling.

The exacerbation of BCS symptoms that occurred because of late diagnosis, liver failure explained by prolonged – PTT, PT, and INR – lower limb edema, ascites, and inadequate medical resources – all these factors combined led to our patient’s unfortunate death. Replacement of the valves may be necessary due to tumor involvement and damage to the mitral and tricuspid valve leaflets^16^.

In a retrospective study^20^ conducted from November 1981 to February 1993, 24 patients with cardiac myxoma were studied; 8 (35%) of the cases were associated with a mitral lesion, and the mitral valve lesion was associated with atrial myxoma. Based on mechanism, the patient can be divided into two groups; one group is due to poor coaptation caused by a tumor above the valve during systole, and the other one is due to the tumor penetrating the valve orifice, causing annular dilation, and directly injuring or destroying the valve component. Sugimoto *et al*.’s^20^ study encourages comprehensive valve examination after myxoma surgical removal, valvuloplasty, and excision of the myxoma to avoid postoperative valvular lesions.

## Conclusion

In conclusion, we have documented a case of large right atrial myxoma-induced supra-hepatic vein thrombosis and BCS, which is a potentially curable etiology of BCS. In the differential diagnosis of BCS, right atrial tumors, including myxomas, should be considered, especially when other explanations are absent.

## Ethical approval

This study is a case report, and the ethical committees in Mauritania do not require ethical approval for such research, but they require obtaining the consent of the patient and the doctor supervising the case.

## Consent

Written informed consent was obtained from the patient for the publication of this case report and accompanying images. A copy of the written consent is available for review by the Editor-in-Chief of this journal on request.

## Sources of funding

There are no funding sources.

## Author contribution

All authors were involved in this work’s conception and design, writing of the paper, article revision, and final revision and approval.

## Conflicts of interest disclosure

The authors declare that they have no conflicts of interest.

## Provenance and peer review

Not commissioned, externally peer-reviewed.
